# Sex difference in the association of the triglyceride glucose index with obstructive coronary artery disease

**DOI:** 10.1038/s41598-023-36135-y

**Published:** 2023-06-14

**Authors:** Ya-Wen Lu, Chuan-Tsai Tsai, Ruey-Hsin Chou, Yi-Lin Tsai, Chin-Sung Kuo, Po-Hsun Huang, Shing-Jong Lin

**Affiliations:** 1 Cardiovascular Center, Taichung Veterans General Hospital, Taichung, Taiwan; 2 Division of Cardiology, Department of Medicine, Taipei Veterans General Hospital, No. 201, Sec. 2, Shih-Pai Road, Taipei, Taiwan; 3 Cardiovascular Research Center, National Yang Ming Chiao Tung University, Taipei, Taiwan; 4 Institute of Clinical Medicine, National Yang Ming Chiao Tung University, Taipei, Taiwan; 5 Department of Critical Care Medicine, Taipei Veterans General Hospital, Taipei, Taiwan; 6 Division of Endocrinology and Metabolism, Department of Medicine, Taipei Veterans General Hospital, Taipei, Taiwan; 7 Department of Medical Research, Taipei Veterans General Hospital, Taipei, Taiwan; 8 Taipei Heart Institute, Taipei Medical University, Taipei, Taiwan; 9 Division of Cardiology, Heart Center, Cheng-Hsin General Hospital, Taipei, Taiwan

**Keywords:** Medical research, Cardiology, Endocrinology, Endocrine system and metabolic diseases, Risk factors

## Abstract

Insulin resistance (IR) is associated with cardiovascular disease in non-diabetic patients. The triglyceride-glucose (TyG) index, incorporating serum glucose and insulin concentrations, is a surrogate insulin resistance marker. We investigated its association with obstructive coronary artery disease (CAD) and sex differences therein. Patients with stable angina pectoris requiring invasive coronary angiography between January 2010 and December 2018 were enrolled. They were divided into two groups according to TyG index. Two interventional cardiologists diagnosed obstructive CAD by angiography review. Demographic characteristics and clinical outcomes were compared between groups. Relative to lower index, patients with higher (≥ 8.60) TyG index had higher BMIs and more prevalent hypertension, diabetes, and elevated lipid profiles [total cholesterol, low-density lipoprotein (LDL), high-density lipoprotein (HDL), triglycerides (TG), fasting plasma glucose (FPG)]. Higher TyG index increased women’s obstructive CAD risk after multivariate adjustment (adjusted odds ratio (aOR) 2.15, 95% confidence interval (95% CI) 1.08–4.26, *p* = 0.02) in non-diabetic populations compared with men. No sex difference was found for diabetic patients. Higher TyG index significantly increased the obstructive CAD risk, overall and for non-diabetic women. Larger-scale studies are needed to confirm our findings.

## Introduction

Cardiovascular disease (CVD) is one of the leading causes of death globally^1^ and the second leading cause of death in Taiwan^2^. Potentially modifiable risk factors for atherosclerotic cardiovascular disease (ASCVD) were obesity, smoking, dyslipidemia, hypertension, diabetes, and insulin resistance^3–5^. IR was related to an increased CVD risk in patients without diabetes due to the direct consequences of elevated insulin and glucose concentrations and pro-coagulant properties^6,7^. IR, directly or indirectly due to associated dyslipidemia, HTN, and chronic inflammation, accelerates atherosclerosis^8^.

The homeostasis model assessment of IR (HOMA-IR) is a validated and frequently used marker to represent IR by incorporating serum glucose and insulin concentrations^9^. However, serum insulin level was not widely measured, limiting its application in clinical practice. Triglyceride-glucose (TyG) index was also a valuable marker of IR with a close relationship between HOMA-IR^10^. Clinical studies revealed that a higher TyG index was associated with increased arterial stiffness/calcification and the progression of coronary atherosclerosis^11–13^. Tyg index is an independent risk factor for major adverse cardiovascular events (MACE) in acute coronary syndrome^14,15^, and chronic coronary syndrome (CCS)^16^.

Type 2 diabetes mellitus (T2DM) was a more potent risk factor for ischemic heart disease (IHD) in women than in men^9,17,18^. Besides, gender discrepancy existed in the distribution of dysglycemia, and impaired glucose tolerance was more prevalent in females^19^. High TyG indices are associated with coronary artery disease (CAD) in patients with T2DM. Still, clinical investigations of sex differences in the impact of such an index on obstructive CAD are scarce. The present study aimed to investigate the implications of higher TyG indices on obstructive CAD in men and women.

## Results

### Baseline characteristics

A total of 720 patients (66.9% men; mean age 69.14 ± 11.94 years) with stable angina pectoris underwent invasive coronary angiography were enrolled. Baseline patient characteristics are shown in Table 1. Compared with the higher TyG index group, patients with lower TyG index were older, less often of the male gender, with lower BMI, and had a lower prevalence of tobacco smoking, HTN, and beta-blockade usage. Higher BMI, the prevalence of DM, and lipid profiles including T-chol, LDL, TG, and FPG, but lower HDL were noticed in the higher TyG index group. Daily medications such as antiplatelet agents, angiotensin-converting enzyme inhibitors, angiotensin II receptor blockers, beta-blockers, calcium channel blockers, diuretics, and statins were similar in the two groups. Among the patients who had obstructive coronary artery disease, 34 patients (10.2%) did not do PCI on the same date of coronary angiography (CAG), 280 (83.8%) did PCI on the same day as CAG, and 19 patients (5.9%) received bypass surgery.

**Table 1:** Baseline characteristics stratified by TyG index in the enrolled patients.

	Total cases n = 720	TyG index < 8.60 n = 360	TyG index ≧8.60 n = 360	*p* value
Age (years)	66.14 ± 11.94	67.74 ± 12.06	64.54 ± 11.63	< 0.001
Male (%)	482 (66.9)	227 (63.1)	255 (70.8)	0.032
Body mass index (kg/m^2^)	25.88 ± 4.17	24.91 ± 3.97	26.84 ± 4.14	< 0.001
Smoking (%)	234 (32.5)	99 (27.5)	135 (37.5)	0.005
Underlying disease				
Hypertension (%)	475 (66.0)	22 (61.9)	252 (70.0)	0.028
Stroke (%)	40 (5.6)	15 (4.2)	25 (7.0)	0.107
Congestive heart failure (%)	54 (7.5)	30 (8.3)	24 (6.7)	0.480
DM (%)	221 (30.7)	67 (18.6)	154 (42.8)	< 0.001
Medication				
Antiplatelet agents (%)	378 (52.5)	182 (50.6)	196 (54.4)	0.332
ACEI or ARB (%)	204 (28.3)	91 (25.3)	113 (31.4)	0.082
Beta blockers (%)	176 (24.4)	71 (19.7)	105 (29.2)	0.004
Calcium channel blockers (%)	170 (23.6)	87 (24.2)	83 (23.1)	0.792
Diuretics (%)	66 (9.2)	32 (8.9)	34 (9.4)	0.897
Statin (%)	230 (31.9)	113 (31.4)	117 (32.5)	0.811
**Laboratory data**				
Total cholesterol (mg/dl)	162.08 ± 32.86	156.15 ± 31.31	168.0 ± 33.34	< 0.001
HDL (mg/dl)	43.44 ± 15.26	47.77 ± 14.15	39.10 ± 15.12	< 0.001
LDL (mg/dl)	95.34 ± 29.15	91.41 ± 27.39	99.25 ± 30.35	< 0.001
Fasting glucose (mg/dl)	110.12 ± 52.34	94.78 ± 17.77	125.46 ± 68.54	< 0.001
Triglyceride (mg/dl)	128.46 ± 83.97	80.18 ± 22.80	176.75 ± 94.47	< 0.001
eGFR (ml/min/1.73m^2^)	66.92 ± 23.72	67.35 ± 21.91	66.49 ± 25.42	0.625
Triglyceride glucose index*	8.67 ± 0.63	8.18 ± 0.31	9.15 ± 0.48	< 0.001
Catheterization finding				
Obstructive CAD	334 (46.4)	150 (41.7)	184 (51.1)	0.013
Single vessel disease	90 (12.5)	46 (12.8)	44 (12.2)	
Double vessel disease	101 (14.0)	49 (13.6)	52 (14.4)	
Triple vessel disease	143 (19.9)	55 (15.3)	88 (24.4)	
Revascularization strategies for obstructive CAD				
PCI	280 (83.8)	128 (85.3)	152 (82.6)	0.386
Bypass surgery	19 (5.7)	6 (4.0)	13 (7.1)	
Medication	35 (10.5)	16 (4.4)	19 (10.3	

Values are mean ± standard deviation or *n* (%).

*DM* diabetes mellitus, *ACEI* angiotensin converting enzyme inhibitor, *ARB* angiotensin II receptor blocker, *HDL* high density lipoprotein, *LDL* low density lipoprotein, *eGFR* estimated glomerular filtration rate, *CAD* coronary artery disease, *PCI* percutaneous coronary intervention.

*Triglyceride glucose index = ln[fasting TG (mg/dL) × fasting plasma glucose (mg/dL)/2].

We stratified the subjects to diabetes or not between gender. For patients with no diabetes, as Table 2 showed, male and female patients with higher TyG index had significantly higher BMI, serum T-chol, LDL, and less HDL level than the lower TyG index group (all p-value < 0.05). Only female patients had a higher prevalence of HTN in the higher TyG index group (lower vs. higher, 48.7% vs. 65.6%, p-value: 0.039). In male patients, higher congestive heart failure prevalence was observed in the lower TyG index group (10.6% vs. 1.4%, p-value: 0.001). In women without diabetes, higher TyG index patients had a greater prevalence of obstructive CAD than the lower TyG index group (19.5% vs. 37.7%, p-value: 0.011). There was no difference in most obstructive CAD in male patients between lower and higher TyG index. Supplement Table S1 showed baseline characteristics of patients with diabetes, stratified by lower and higher TyG index. Only significantly higher serum T-chol and LDL levels were found in female patients with a higher TyG index. Male patients with higher TyG index were younger, more obese, and had higher T-chol and LDL levels compared with lower TyG index patients. There was no difference in obstructive CAD prevalence between sex stratified by lower and higher TyG index.

**Table 2:** Baseline characteristics in non-diabetic patients with lower and higher TyG index stratified by sex.

	Non-DM Female (n = 174)			Non-DM Male (n = 325)		
	TyG index < 8.60 n = 113	TyG index ≧8.60 n = 61	p value	TyG index < 8.60 n = 180	TyG index ≧8.60 n = 145	p value
Age (years)	64.96 ± 10.37	65.07 ± 10.54	0.951	68.67 ± 13.32	63.86 ± 11.57	0.001
Body mass index (kg/m^2^)	24.50 ± 3.66	26.07 ± 3.24	0.006	24.91 ± 4.15	26.62 ± 3.53	< 0.001
Smoking (%)	11 (9.7)	5 (8.2)	1.000	69 (38.3)	72 (49.7)	0.043
Underlying disease						
Hypertension (%)	55 (48.7)	40 (65.6)	0.039	110 (61.1)	88 (60.7)	1.000
Stroke (%)	2 (1.8)	2 (3.3)	0.610	10 (5.6)	12 (8.3)	0.378
Congestive heart failure (%)	5 (4.4)	4 (6.6)	0.721	19 (10.6)	2 (1.4)	0.001
Medication						
Antiplatelet agents (%)	48 (42.5)	21 (34.4)	0.333	94 (52.2)	83 (57.2)	0.373
ACEI or ARB (%)	19 (16.8)	16 (26.2)	0.166	45 (25.0)	43 (29.7)	0.380
Beta blockers (%)	24 (21.2)	14 (23.0)	0.848	35 (19.4)	39 (26.9)	0.143
Calcium channel blockers (%)	18 (15.9)	13 (21.3)	0.410	40 (22.2)	28 (19.3)	0.584
Diuretics (%)	7 (6.2)	5 (8.2)	0.755	15 (8.3)	11 (7.6)	0.840
Statin (%)	32 (28.3)	11 (18.0)	0.145	55 (30.6)	47 (32.4)	0.720
Laboratory data						
Total cholesterol (mg/dl)	165.54 ± 27.90	181.97 ± 34.90	0.001	154.64 ± 33.04	170.72 ± 33.17	< 0.001
HDL (mg/dl)	52.87 ± 13.35	46.93 ± 16.30	0.011	45.31 ± 13.46	36.62 ± 14.60	< 0.001
LDL (mg/dl)	94.67 ± 27.04	104.97 ± 32.68	0.035	91.99 ± 27.77	103.88 ± 31.69	0.001
Fasting glucose (mg/dl)	91.90 ± 14.92	106.80 ± 25.31	< 0.001	93.82 ± 16.43	112.83 ± 90.18	0.006
Triglyceride (mg/dl)	81.99 ± 22.08	171.23 ± 82.79	< 0.001	79.50 ± 24.02	186.72 ± 97.51	< 0.001
Uric acid (mg/dl)	5.35 ± 1.69	5.30 ± 1.57	0.864	6.52 ± 3.23	6.59 ± 1.50	0.810
eGFR (ml/min/1.73m^2^)	71.93 ± 21.30	71.10 ± 22.16	0.809	67.55 ± 21.16	70.19 ± 19.20	0.245
Triglyceride glucose index*	8.19 ± 0.29	9.03 ± 0.38	< 0.001	8.16 ± 0.32	9.10 ± 0.46	< 0.001
Catheterization finding						
Obstructive CAD	22 (19.5)	23 (37.7)	0.011	88 (48.9)	72 (49.7)	0.911
Single vessel disease	11 (9.7)	4 (6.6)	0.002	25 (13.9)	23 (15.9)	0.940
Double vessel disease	7 (6.2)	7 (11.5)		28 (15.6)	20 (13.8)	
Triple vessel disease	4 (3.5)	12 (19.7)		35 (19.4)	29 (20.0)	

Values are mean ± standard deviation, *n* (%).

*DM* diabetes mellitus, *ACEI* angiotensin converting enzyme inhibitor, *ARB* angiotensin II receptor blocker, *HDL* high density lipoprotein, *LDL* low density lipoprotein, *eGFR* estimated glomerular filtration rate, *CAD* coronary artery disease.

*Triglyceride glucose index = ln[fasting TG (mg/dL) × fasting plasma glucose (mg/dL)/2].

**Figure 1:**
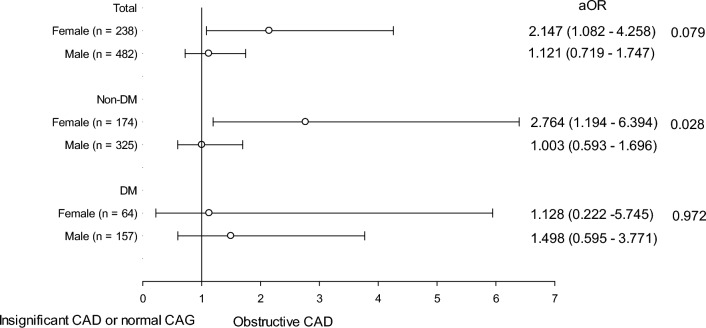
Gender disparity in enrolled patients and separated to non-DM & DM. *aOR* adjusted odds ratio, variables adjusted: age, BMI, smoking, HTN, CHF, stroke, LDL, eGFR, statin, antiplatelet agents, angiotensin converting enzyme inhibitor or angiotensin II receptor blocker.

The multivariate logistic regression analysis of all patients in supplement Table S2. Revealed a higher TyG index independently associated with obstructive CAD (adjusted odds ratio (aOR): 1.402, 95% CI 1.002–1.961, p-value: 0.049). Table 3 demonstrates the gender disparity. In multivariate logistic regression analysis of females without diabetes, higher TyG index (adjusted odds ratio (aOR): 2.568, 95% CI 1.238–5.327, p value:0.011), CHF (aOR: 4.862, 95% CI 1.050–22.508, p-value: 0.043) and eGFR (aOR: 0.980, 95% CI: 0.964–0.996, p-value: 0.016) independently related to obstructive CAD. For men without diabetes, the use of antiplatelet agents (aOR: 2.162, 95% CI 1.320–3.542, p-value: 0.002), statins (aOR: 2.678, 95% CI: 1.555–4.612, p-value: < 0.001) and eGFR (aOR: 0.987, 95% CI: 0.974–1.000, p-value: 0.054) were independently associated with obstructive CAD. Figure 1 revealed the impact of a higher TyG index in obstructive CAD between enrolled patients, p interaction in patients without diabetes was 0.046 after adjusting the conventional CVD risk factors.

**Table 3:** Univariate and multivariate logistic regression analysis of factors associated with the incidence of obstructive CAD in non-DM patients (n = 499).

Variable	Univariate analysis			Multivariate analysis		
	OR	95% CI	*p* value	OR	95% CI	*p* value
Female (n = 174)						
Higher TyG index	2.504	1.248–5.023	0.010	2.568	1.238–5.327	0.011
Age (years)	1.046	1.009–1.084	0.014	1.027	0.987–1.068	0.197
Body mass index (kg/m^2^)	1.014	0.922–1.116	0.767			
Smoking (%)	1.341	0.439–4.094	0.607			
Hypertension (%)	2.249	1.095–4.617	0.027	1.453	0.665–3.176	0.348
Stroke (%)	0.947	0.096–9.343	0.963			
Congestive heart failure (%)	6.462	1.544–27.046	0.011	4.862	1.050–22.508	0.043
Antiplatelet agents (%)	1.477	0.744–2.932	0.265			
ACEI or ARB (%)	1.676	0.753–3.729	0.206			
Beta blockers (%)	1.690	0.7760–3.681	0.187			
Calcium channel blockers (%)	1.216	0.513–2.881	0.657			
Diuretics (%)	2.179	0.655–7.247	0.204			
Statin (%)	1.804	0.854–3.810	0.122			
Total cholesterol (mg/dl)	1.006	0.995–1.017	0.295			
HDL (mg/dl)	0.982	0.958–1.007	0.150			
Uric acid	1.164	0.952–1.423	0.138			
eGFR (ml/min/1.73m^2^)	0.978	0.963–0.993	0.005	0.980	0.964–0.996	0.016
Male (n = 325)						
Higher TyG index	1.031	0.666–1.597	0.891			
Age (years)	1.024	1.007–1.042	0.007	1.015	0.994–1.037	0.162
Body mass index (kg/m^2^)	1.009	0.955–1.067	0.737			
Smoking (%)	0.932	0.601–1.445	0.751			
Hypertension (%)	2.031	1.289–3.199	0.002	1.338	0.803–2.230	0.264
Stroke (%)	1.533	0.636–3.693	0.341			
Congestive heart failure (%)	1.144	0.472–2.774	0.765			
Antiplatelet agents (%)	2.890	1.837–4.545	< 0.001	1.949	1.164–3.266	0.011
ACEI or ARB (%)	1.339	0.820–2.188	0.244			
Beta blockers (%)	2.291	1.338–3.925	0.003	1.587	0.870–2.896	0.132
Calcium channel blockers (%)	1.300	0.761–2.223	0.337			
Diuretics (%)	1.034	0.464–2.305	0.935			
Statin (%)	3.448	2.092–5.685	< 0.001	2.631	1.508–4.590	0.001
Total cholesterol (mg/dl)	0.994	0.987–1.000	0.068	0.999	0.992–1.007	0.844
HDL (mg/dl)	1.002	0.987–1.017	0.806			
Uric acid (mg/dl)	0.986	0.903–1.076	0.744			
eGFR (ml/min/1.73m^2^)	0.981	0.970–0.993	0.001	0.987	0.973–1.000	0.058

*ACEI* angiotensin converting enzyme inhibitor, *ARB* angiotensin II receptor blocker, *HDL* high-density lipoprotein, *eGFR* estimated glomerular filtration rate.

## Discussion

Our results showed that a higher TyG index (TyG index≧8.60), as the surrogate marker of insulin resistance, was significantly associated with obstructive CAD compared with a lower TyG index (TyG index < 8.60). Besides, the impact of a higher TyG index and the risk of obstructive CAD is prominent in women but not men without diabetes.

### The impact of insulin resistance and CVD

IR accelerates atherosclerosis and increases the incidence of CVD due to chronic inflammation or pro-coagulant properties. IR may precede type 2 DM for several years and is associated with various cardiometabolic risk factors like dyslipidemia, hyperglycemia, obesity, and hypertension contributing to further CVD development^7,8^.

TyG index had been reported as the surrogate marker of IR in 2008 for large cross-sectional healthy populations^10^. Several studies examined the TyG index as a predictor factor in metabolic disorders, acute coronary syndrome (ACS), or nonalcoholic fatty liver disease (NAFLD) subjects. Elevated TyG index is significantly associated with a higher risk of arterial stiffness^11,20^, further diabetes development^21^, and progression of coronary artery calcification^22^. Besides, a higher TyG index is also an independent risk factor of MACE in the ACS population^15,23^. Our study supported the previous work that a higher TyG index significantly increases the risk of obstructive CAD and extends the notion that females may experience a greater risk of CAD if TyG is elevated than males.

### Gender difference in CHD risk and insulin resistance

Women tend to present later CHD than men in life according to the large prospective observational longitudinal registry of patients with stable coronary artery disease (CLARIFY) study reported in 2012. Women with chronic stable angina were more likely to be older and have co-morbidities such as hypertension and diabetes mellitus^24^. The clinical presentation was more atypical in women than men, which may mislead life-threatening diseases such as CAD and delay effective treatment such as reperfusion therapy^25,26^. Previous studies also revealed that women were associated with poor prognosis in ST-segment elevation myocardial infarction (STEMI) than men after reperfusion therapy^27,28^. TyG index may provide additional clues apart from the control of co-morbidities such as hypertension and DM to prevent CAD in the female gender.

In the present study, a higher TyG index in non-diabetic women was significantly associated with a higher risk of obstructive CAD than non-diabetic men. However, the gender difference was not found in the diabetic group. Previous studies have demonstrated that diabetes precipitates other CVD risk factors by changes in the coagulation, inflammation, and fibrinolytic system in women than in men^29^. That partially explained by the difference in insulin resistance (HOMA-IR) and more central adiposity between diabetic and non-diabetic women than men^29,30^.

To transition from normoglycemia to diabetes, women passed through adverse metabolic disturbances more than men^31,32^. Women experience much change in rates of BMI and deteriorations of lipid profile compared to men even when cardiovascular risk factors (CVRF) are similar^33^. During insulin resistance, impaired nitric oxide (NO) secretion and the overproduction of reactive oxygen species by endothelial cells lead to CVD development^34,35^. In the mice model, the endothelial dysfunction was severe in female hypertriglyceridemic rats compared to males (HTGs)^36^. Estrogen preserves the endothelial function, as confirmed by the research conducted in humans and mice. Women with polycystic ovary syndrome were found to have altered endothelial function due to elevated serum androgen levels and increased insulin resistance^37^. Chronic estrogen supplement in insulin-treated ovariectomized Wistar rats prevents insulin-induced vasoconstriction^38^. When glucose tolerance deteriorates towards type 2 diabetes, that kind of gender difference gradually accounted less important with a similar extent of insulin resistance^39,40^. Estrogen deficiency and IR probably increase the risk of CAD development; therefore, the TyG index, as the surrogate marker of insulin resistance in non-diabetic women, requires further investigation.

### Study limitations

The limitations of our study were as follows. First, some confounding factors remained unmeasured due to the small sample size (n = 720) and the retrospective, observational study design in a single medical center. Second, the potential bias might be that fewer female patients did the invasive catheterization than male patients. Females who performed invasive coronary angiography may have more severe symptoms or abnormal non-invasive studies. Third, we only separated CAD as obstructed or not but lack of data about disease severity (e.g.,degree of vessel stenosis or plaque burden). Thus, whether higher TyG index related to CAD severity such as Syntax score remained unknown. Fourth, the impact of TyG index between sexes in different ethnicity needs further investigation because only Asians enrolled in our study. Fifth, there was no serum insulin data in the present study; therefore, another method to evaluate insulin resistance, like HOMA-IR deserves other studies for clarification.

## Conclusion

In this retrospective cohort study, we found that a higher TyG index was significantly increased the risk of obstructive CAD with gender disparities in non-diabetic patients after adjusting other traditional risk factors of CAD. Further larger-scale studies are needed to confirm these findings.

## Materials

### Study design and population

In this cross-sectional observational study, consecutive patients with stable angina pectoris and abnormal non-invasive tests, including exercise electrocardiography, nuclear test, or coronary computed tomography angiography (CTA) indicated coronary angiography between January 2010 and December 2018, were enrolled. Patients with the following conditions were excluded: (1) age < 30 years, (2) combined with peripheral catheterization, (3) with missing data of glucose or triglyceride, and (4) refusal of clinical follow-up. All participants provided written informed consent. The study was approved by the research ethics committee of the Taipei Veterans General Hospital (Ethics approval registration number: 2012-03-001AC) and was conducted in accordance with the Declaration of Helsinki.

### Demography and laboratory examinations

**Figure 2:**
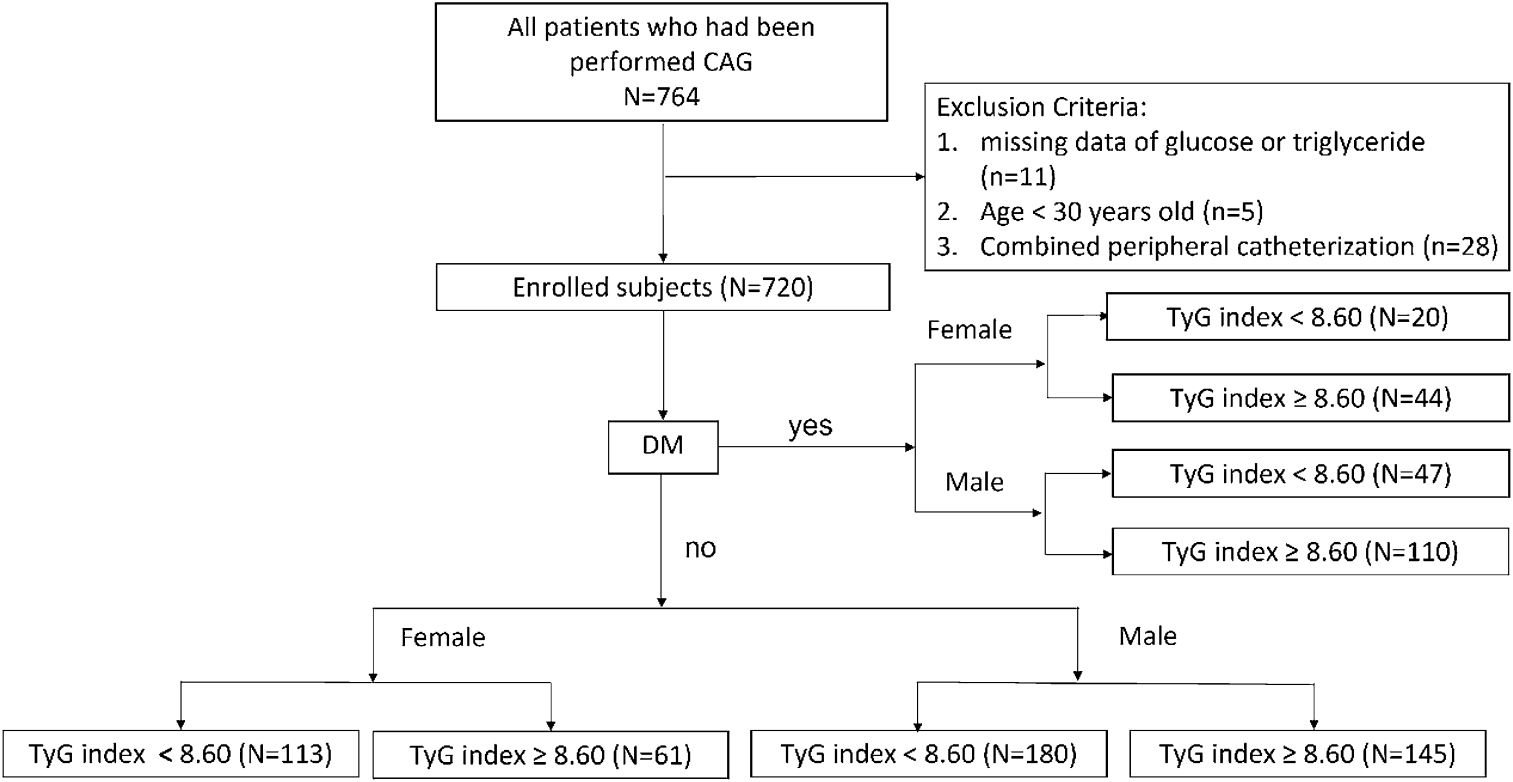
Flow chart for patients enrollment.

The past medical history was recorded through a detailed chart review. Questionnaire was also provided for those subjects that medical records were not available at our hospital, including history of chronic diseases and medications. All subjects received anthropometric measurements by research nurses, including assessments of height, weight, and blood pressure. BMI was calculated as weight (in kilograms) divided by height (in meters) squared. Blood samples were obtained from all subjects after overnight fasts of ≥ 8 h. Serum levels of creatinine, total cholesterol, low density lipoprotein, high density lipoprotein, triglyceride and glucose were measured using an automated analyzer (AVDIA 1800; Siemens, Malvern, PA, USA) in a colorimetric assay. The eGFR was calculated by using the Chronic Kidney Disease Epidemiology Collaboration equation^41^. TyG index was calculated as the formula: ln[fasting TG (mg/dL) × fasting plasma glucose (mg/dL)/2]^10^. Patients were divided into two equal groups according to the median TyG index. The flowchart of patient enrollment and classification was illustrated in Fig. 2.

Obstructive coronary artery disease (CAD) was defined as any stenosis 50% or greater in the left main coronary artery, and 70% or greater in any other coronary artery^42,43^. Percutaneous coronary artery intervention (PCI) was performed as clinically indicated; both bare-metal and drug-eluting stents were used for revascularization.

### Statistical analysis

Data were expressed as frequencies (percentages) for categorical variables and as means ± standard deviations for continuous variables. The Chi-square test was used for comparisons of categorical variables, and the independent t-test was employed for continuous variables. Logistic regression analysis was performed to assess the relationships between higher TyG index and obstructive CAD. Subgroup analysis was conducted to investigate the association of higher TyG index to obstructive CAD stratified by different gender in the whole cohort, DM, and non-DM populations. Multinomial logistic regression analyses were performed in order to assess the association of TyG index with obstructive CAD after adjustment for confounding factors. The confounding factors included age, sex, smoking history, hypertension, DM, previous stroke, LDL-C, eGFR, statin use, antiplatelet agents use, ACEI/ARB use and TyG index. Odds ratios with 95% confidence intervals (95% CI) for the risk of obstructive CAD are reported. Statistical analyses were performed using SPSS version 23.0 (SPSS, version 24.0.0.0, IBM Corporation, Armonk, New York, USA). Two-tailed *p* values < 0.05 were regarded as statistically significant.

## Supplementary Information

Supplementary Table 1.

Supplementary Table 2.

## Data Availability

The datasets generated during and/or analysed during the current study are available from the corresponding author on reasonable request.
