# Supplementary material for: Sex difference in the association of the triglyceride glucose index with obstructive coronary artery disease
Source: Sci Rep. 2023 Jun 14;13:9652. doi: 10.1038/s41598-023-36135-y (PMC10267172; doi:10.1038/s41598-023-36135-y)
Supplement: Supplementary file 1 — Supplementary Table 1. [file 41598_2023_36135_MOESM1_ESM.docx]

**Supplement Table 1. Baseline characteristics in diabetic patients with lower and higher TyG index stratified by sex**

|  | **DM Female (n=64)** | | | **DM Male (n=157)** | | |
| --- | --- | --- | --- | --- | --- | --- |
|  | **TyG index < 8.60**  **n=20** | **TyG index ≧8.60**  **n=44** | **p value** | **TyG index < 8.60**  **n=47** | **TyG index ≧8.60**  **n=110** | **p value** |
| Age (years) | 67.40 ± 10.41 | 66.86 ± 10.59 | 0.851 | 70.96 (63-79) | 64.21 (55-72.5) | 0.001 |
| Body mass index (kg/m^2^) | 26.41 ± 4.59 | 27.56 ± 6.32 | 0.471 | 25.24 (22.62-27.64) | 27.28 (24.66-29.09) | 0.005 |
| Smoking (%) | 1 (5.0) | 2 (4.5) | 1.000 | 18 (38.3) | 56 (50.9) | 0.165 |
| **Underlying disease** |  |  |  |  |  |  |
| Hypertension (%) | 16 (80.0) | 34 (77.3) | 1.000 | 42 (89.4) | 90 (81.8) | 0.341 |
| Stroke (%) | 1 (5.0) | 3 (6.8) | 1.000 | 2 (4.3) | 8 (7.3) | 0.724 |
| Congestive heart failure (%) | 3 (15.0) | 7 (15.9) | 1.000 | 3 (6.4) | 11 (10.0) | 0.556 |
| **Medication** |  |  |  |  |  |  |
| Antiplatelet agents (%) | 7 (35.0) | 26 (59.1) | 0.106 | 33 (70.2) | 66 (60.0) | 0.280 |
| ACEI or ARB (%) | 7 (35.0) | 20 (45.5) | 0.586 | 20 (42.6) | 34 (30.9) | 0.199 |
| Beta blockers (%) | 4 (20.0) | 15 (34.1) | 0.377 | 8 (17.0) | 37 (33.6) | 0.036 |
| Calcium channel blockers (%) | 10 (50.0) | 11 (25.0) | 0.083 | 19 (40.4) | 31 (28.2) | 0.139 |
| Diuretics (%) | 1 (5.0) | 9 (20.5) | 0.152 | 9 (19.1) | 9 (8.2) | 0.059 |
| Statin (%) | 6 (30.0) | 10 (22.7) | 0.547 | 20 (42.6) | 49 (44.5) | 0.862 |
| OHA (%) | 9 (45.0) | 23 (52.3) | 0.788 | 31 (67.4) | 62 (57.4) | 0.283 |
| insulin | 1 (5.0) | 13 (29.5) | 0.047 | 5 (10.9) | 20 (18.5) | 0.340 |
| **Laboratory data** |  |  |  |  |  |  |
| Total Cholesterol (mg/dl) | 146.15 (128.25-169.75) | 170.70 (150-196) | 0.005 | 143.68 ± 26.40 | 155.65 ± 29.17 | 0.017 |
| HDL (mg/dl) | 51.14 ± 16.38 | 44.31 ± 17.98 | 0.156 | 43.42 (33.85-49.4) | 35.95 (29-41.6) | <0.001 |
| LDL (mg/dl) | 77.90 (59.5-103.75) | 98.08 (83.25-118) | 0.009 | 87.41 ± 25.73 | 90.28 ± 26.42 | 0.556 |
| Fasting glucose (mg/dl) | 94.45 (78.25-111.25) | 156.84 (114.5-170.25) | <0.001 | 105.51 (92-119) | 139.89 (109-165.25) | <0.001 |
| Triglyceride (mg/dl) | 83.50 (65.50-104.0) | 155.95 (100.25-185) | <0.001 | 76.98 (64-89) | 174.99 (114.25-211) | <0.001 |
| Uric acid | 5.72 ± 1.56 | 6.17 ± 1.88 | 0.386 | 6.65 ± 1.75 | 6.02 ± 1.74 | 0.044 |
| eGFR (ml/min/1.73m^2^) | 60.44 ± 29.22 | 51.94 ± 33.21 | 0.239 | 58.51 (42.85-70.91) | 64.87 (46.31-87.76) | 0.052 |
| Triglyceride glucose index* | 8.22 (7.95-8.42) | 9.24 (8.84-9.59) | <0.001 | 8.25 (8.02-8.50) | 0.26 (8.90-9.50) | <0.001 |
| **Catheterization finding** |  |  |  |  |  |  |
| Obstructive CAD | 8 (40.0) | 19 (43.2) | 1.000 | 32 (68.1) | 70 (63.6) | 0.715 |
| Single vessel disease | 4 (20.0) | 4 (9.1) | 0.477 | 6 (12.8) | 13 (11.8) | 0.577 |
| Double vessel disease | 3 (15.0) | 9 (20.5) |  | 11 (23.4) | 16 (14.5) |  |
| Triple vessel disease | 1 (5.0) | 6 (13.6) |  | 15 (31.9) | 41 (37.3) |  |

Values are mean ± standard deviation, *n* (%) or mean (25^th^ percentile-75^th^ percentile). Abbreviations: DM=diabetes mellitus, ACEI = angiotensin converting enzyme inhibitor, ARB = angiotensin II receptor blocker, HDL=high density lipoprotein, LDL = low density lipoprotein, eGFR = estimated glomerular filtration rate, CAD = coronary artery disease.

* Triglyceride glucose index= ln[fasting TG (mg/dL) × fasting plasma glucose (mg/dL)/2]
