# Supplementary material for: Sex difference in the association of the triglyceride glucose index with obstructive coronary artery disease
Source: Sci Rep. 2023 Jun 14;13:9652. doi: 10.1038/s41598-023-36135-y (PMC10267172; doi:10.1038/s41598-023-36135-y)
Supplement: Supplementary file 2 — Supplementary Table 2. [file 41598_2023_36135_MOESM2_ESM.docx]

Supplement Table 2. Cox regression analysis in overall patients (n=720)

|  | **Univariate analysis** | | | **Multivariate analysis** | | |
| --- | --- | --- | --- | --- | --- | --- |
| **Variable** | **OR** | **95% CI** | ***p* value** | **OR** | **95% CI** | ***p* value** |
| Higher TyG index | 1.464 | 1.090-1.964 | 0.011 | 1.402 | 1.002-1.961 | 0.049 |
| Age (years) | 1.028 | 1.015-1.041 | <0.001 | 1.014 | 0.999-1.030 | 0.072 |
| Gender | 2.746 | 1.975-3.818 | <0.001 | 2.647 | 1.837-3.816 | <0.001 |
| Body mass index (kg/m^2^) | 1.004 | 0.969-1.040 | 0.818 |  |  |  |
| Smoking (%) | 1.408 | 1.030-1.925 | 0.032 | 0.980 | 0.669-1.435 | 0.918 |
| Hypertension (%) | 2.503 | 1.811-3.459 | <0.001 | 1.767 | 1.231-2.538 | 0.002 |
| Stroke (%) | 2.237 | 1.148-4.359 | 0.018 | 1.196 | 0.570-2.507 | 0.636 |
| Congestive heart failure (%) | 1.488 | 0.852-2.601 | 0.162 |  |  |  |
| DM | 2.011 | 1.458-2.773 | <0.001 | 1.317 | 0.901-1.924 | 0.155 |
| Total Cholesterol (mg/dl) | 0.992 | 0.987-0.997 | 0.001 | 0.999 | 0.994-1.005 | 0.780 |
| HDL (mg/dl) | 0.985 | 0.975-0.995 | 0.002 | 0.996 | 0.984-1.007 | 0.465 |
| Uric acid | 1.059 | 0.981-1.143 | 0.144 |  |  |  |
| eGFR (ml/min/1.73m^2^) | 0.985 | 0.975-0.995 | 0.002 | 0.984 | 0.977-0.992 | <0.001 |
| Antiplatelet agents (%) | 2.472 | 1.828-3.342 | <0.001 | 1.582 | 1.119-2.236 | 0.009 |
| ACEI or ARB (%) | 0.656 | 0.473-0.908 | 0.011 | 1.146 | 0.767-1.713 | 0.506 |
| Beta blockers (%) | 1.972 | 1.396-2.785 | <0.001 | 1.356 | 0.906-2.030 | 0.138 |
| Calcium channel blockers (%) | 1.411 | 0.999-1.992 | 0.051 | 0.759 | 0.500-1.1523 | 0.195 |
| Diuretics (%) | 1.097 | 0.661-1.821 | 0.720 |  |  |  |
| Statin (%) | 2.717 | 1.966-3.755 | <0.001 | 2.212 | 1.524-3.211 | <0.001 |

Abbreviations: ACEI= angiotensin converting enzyme inhibitor, ARB = angiotensin II receptor blocker; HDL= high-density lipoprotein; eGFR = estimated glomerular filtration rate
